# A forward modeling approach to analyzing galaxy clustering with SimBIG

**DOI:** 10.1073/pnas.2218810120

**Published:** 2023-10-11

**Authors:** ChangHoon Hahn, Michael Eickenberg, Shirley Ho, Jiamin Hou, Pablo Lemos, Elena Massara, Chirag Modi, Azadeh Moradinezhad Dizgah, Bruno Régaldo-Saint Blancard, Muntazir M. Abidi

**Affiliations:** ^a^Department of Astrophysical Sciences, Princeton University, Princeton NJ 08544; ^b^Center for Computational Mathematics, Flatiron Institute, New York, NY 10010; ^c^Center for Computational Astrophysics, Flatiron Institute, New York, NY 10010; ^d^Department of Astronomy, University of Florida, Gainesville, FL 32611; ^e^Max-Planck-Institut für Extraterrestrische Physik, Garching bei München 85748, Germany; ^f^Department of Physics, Université de Montréal, Montréal, QC H2V 0B3, Canada; ^g^Mila - Quebec Artificial Intelligence Institute, Montréal, QC H2S 3H1, Canada; ^h^Waterloo Centre for Astrophysics, University of Waterloo, Waterloo, ON N2L 3G1, Canada; ^i^Department of Physics and Astronomy, University of Waterloo, Waterloo, ON N2L 3G1, Canada; ^j^Département de Physique Théorique, Université de Genève, Genève 4 1211, Switzerland

**Keywords:** cosmology, machine learning, galaxies, simulations

## Abstract

We present cosmological constraints from a simulation-based inference (SBI) analysis of galaxy clustering from the SimBIG forward modeling framework. SimBIG leverages the predictive power of high-fidelity simulations and provides an inference framework that can extract cosmological information on small nonlinear scales. In this work, we apply SimBIG to the Baryon Oscillation Spectroscopic Survey (BOSS) CMASS galaxy sample and analyze the power spectrum, $P_{\ell }\left(k\right)$, to $k_{\max}=0.5\;h/\mathrm{Mpc}$. We construct 20,000 simulated galaxy samples using our forward model, which is based on 2,000 high-resolution QuijoteN-body simulations and includes detailed survey realism for a more complete treatment of observational systematics. We then conduct SBI by training normalizing flows using the simulated samples and infer the posterior distribution of ΛCDM cosmological parameters: $\Omega_{m},\Omega_{b},h,n_{s},\sigma_{8}$. We derive significant constraints on $\Omega_{m}$ and $\sigma_{8}$, which are consistent with previous works. Our constraint on $\sigma_{8}$ is 27% more precise than standard $P_{\ell }$ analyses because we exploit additional cosmological information on nonlinear scales beyond the limit of current analytic models, k>0.25h/Mpc. This improvement is equivalent to the statistical gain expected from a standard $P_{\ell }$ analysis of galaxy sample ∼60% larger than CMASS. While we focus on $P_{\ell }$ in this work for validation and comparison to the literature, SimBIG provides a framework for analyzing galaxy clustering using any summary statistic. We expect further improvements on cosmological constraints from subsequent SimBIG analyses of summary statistics beyond $P_{\ell }$.

## Significance

The three-dimensional spatial distribution of galaxies encodes key cosmological information on the nature of dark energy and the contents of the Universe. Current analyses of the statistical clustering of galaxies successfully extract information on large scales that are well described by analytic models. They, however, struggle on smaller, nonlinear, scales. Here, we present SimBIG, an approach to galaxy clustering analyses that can extract information on nonlinear regimes by exploiting high-fidelity simulations and inference based on machine learning. To demonstrate its advantages, we apply SimBIG to 109,636 galaxies of the BOSS survey and analyze a standard summary statistic of the galaxy distribution. Our constraints are consistent with previous works and substantially improve their precision on select cosmological parameters.

The three-dimensional spatial distribution of galaxies provides key cosmological information that can be used to constrain the nature of dark matter and dark energy and measure the contents of the Universe. The next generation spectroscopic galaxy surveys, conducted using the Dark Energy Spectroscopic Instrument (DESI; 1, 2, 3), Subaru Prime Focus Spectrograph (PFS; 4, 5), the ESA *Euclid* satellite mission (6), and the Nancy Gracy Roman Space Telescope (Roman; 7, 8), will probe galaxies over cosmic volumes out to z∼3, over 10 Gyrs of cosmic history. Combined with other cosmological probes, they will provide the most stringent tests of the standard ΛCDM cosmological model and potentially lead to discoveries of new physics.

In current analyses, the power spectrum is used as the primary measurement of galaxy clustering (e.g., refs. 9, 10, 11, 12, 13, 14, 15). Furthermore, the analyses are limited to large, linear scales where the impact of nonlinear structure formation is small. These restrictions result from the fact that standard analyses use analytic models based on perturbation theory (PT) of large-scale structure, see refs. 16 and 17 for a review. PT struggles to accurately model scales beyond quasi-linear scales, especially for higher-order clustering statistics (e.g., bispectrum). In the (18) PT analysis, for instance, the authors restrict the power spectrum to k<0.2h/Mpc and the bispectrum to k<0.08h/Mpc. In a recent PT analysis, (19) analyzes the bispectrum to k<0.23h/Mpc; however, they require 33 extra parameters for the theoretical consistency of their model. PT also cannot be used to model various recently proposed summary statistics, e.g., ref. 20 or to exploit the full galaxy distribution at the field level. While some analyses in real space have analyzed galaxy clustering on smaller scales, e.g., refs. 21–24, they do not simultaneously analyze clustering on large scales.

Another major challenge for current analyses is accurately accounting for observational systematics. Observations suffer from imperfections in, e.g., targeting, imaging, and completeness that can significantly impact the analysis (25, 26). Current analyses account for these effects by applying correction weights to the galaxies. Fiber collisions, for example, prevent galaxy surveys that use fiber-fed spectrographs (e.g., DESI, PFS) from successfully measuring redshifts from galaxies within some angular scale of one another on the focal plane. This significantly biases the power spectrum by more than the amplitude of cosmic variance on scales smaller than k>0.1h/Mpc (27–29). To correct for this effect, the weights of the “collided” galaxies missed by survey are assigned to their nearest angular neighbors (30, 31). Even for current analyses, these correction weights do not sufficiently correct the measured power spectrum (28). Furthermore, they are only designed and demonstrated for the power spectrum.

Meanwhile, additional cosmological information is available on nonlinear scales and in higher-order statistics. Recent studies have accurately quantified the information content in these regimes using large suites of simulations. (32) and (33) used the Quijote suite of simulations to demonstrate that constraints on cosmological parameters, $\Omega_{m},\Omega_{b},h,n_{s},\sigma_{8}$, improve by a factor of ∼2 by including nonlinear scales (0.2<k<0.5h/Mpc) in power spectrum analyses. Even more improvement comes from including higher-order clustering information in the bispectrum. Similar forecasts for other summary statistics, e.g., marked power spectrum (34, 35), reconstructed power spectrum (36), skew spectra (37), wavelet statistics (38), find consistent improvements from including nonlinear scales and higher-order clustering. Despite the growing evidence of the significant constraining power available in nonlinear scales and higher-order statistics, it cannot be exploited by standard methods with PT.

Robustly exploiting nonlinear and non-Gaussian cosmological information requires a framework that can both accurately model nonlinear structure formation and account for detailed observational systematics. In this work, we present SIMulation-Based Inference of Galaxies (SimBIG), a framework for analyzing galaxy clustering that achieves these requirements by using a forward modeling approach. Instead of analyzing galaxy clustering using analytic models, a forward model approach uses simulations that model the full details of the observations.

In SimBIG, our forward model is based on cosmological N-body simulations that accurately model nonlinear structure formation. We also use the halo occupation framework, which provides a compact and flexible prescription for connecting the galaxy distribution to the dark matter distribution. Our forward model also takes advantage of the fact that many observational systematics can be more easily simulated, e.g., refs. 39 and 40 than corrected in the observations. With this forward model, we can rigorously analyze galaxy clustering on nonlinear scales and with higher-order statistics.

To infer the cosmological parameters, our approach does not require sampling the posterior using an assumed analytic likelihood. We instead use simulation-based inference SBI; see ref. 41, for a review. SBI, also known as “likelihood-free inference,” enables accurate Bayesian inference using forward models, e.g., refs. 42–45. Moreover, they leverage neural density estimation from machine learning, e.g., refs. 42 and 46 to more efficiently infer the posterior without sampling or making strong assumptions on the functional form of the likelihood.

In this work, we apply SimBIG to the CMASS galaxy sample observed by the Sloan Digital Sky Survey SDSS-III Baryon Oscillation Spectroscopic Survey (BOSS; 47, 48). With the main goal of demonstrating the accuracy and potential of SimBIG, we use the power spectrum as our summary statistic. We present the cosmological constraints inferred from our analysis and compare them to previous constraints in the literature. In an accompanying paper (49, hereafterH22b), we present our forward model in further detail and the mock challenge that we conduct to rigorously validate the accuracy and precision of SimBIG cosmological constraints.

## Simulation-Based Inference of Galaxies SIMBIG

Modern cosmological analyses use Bayesian inference to constrain the posterior distribution p(θ|x) of cosmological parameters, θ, given observation, x. In standard galaxy clustering analyses, the posterior is evaluated using Bayes’ rule. The likelihood is assumed to have a Gaussian functional form and evaluated using an analytic PT model.

SBI offers an alternative that requires no assumptions on the form of the likelihood. SBI only requires a forward model—i.e., a simulation that can generate a realization of mock observations, $x^{\prime }$, given set of parameters, $\theta^{\prime }$. Each realization $x^{\prime }$ corresponds to a sample drawn from the likelihood $p(x\;|\;\theta^{\prime })$. It uses a training dataset of simulated pairs $\left\{\left(\theta^{\prime },x^{\prime }\right)\right\}$ to estimate the posterior. SBI has already been successfully applied to a wide range of inference problems in astronomy and cosmology (43, 44, 50–55).

In this work, we utilize SBI based on neural density estimation, where a neural network q with parameters ϕ is trained to estimate $p\left(\theta \;|\;x\right)\approx q_{\phi }\left(\theta \;|\;x\right)$. In particular, we use “normalizing flow” models that are capable of accurately estimating complex distributions highly efficiently (56, 57). Below, we briefly describe our forward model and SBI framework.

### A. Forward Model.

SBI requires a forward model capable of generating mock observations that are statistically indistinguishable from the observations. We start with high-resolution N-body simulations from the Quijote suite (58). These simulations follow the evolution of $1024^{3}$ cold dark matter (CDM) particles in a volume of ${\left(1\;h^{-1}\mathrm{Gpc}\right)}^{3}$ from z=127 to z=0.5 using the TreePM GADGET-III code. They accurately model the clustering of matter down to nonlinear scales beyond k=0.5h/Mpc (58).

To model the galaxy distribution, we identify gravitationally bound dark matter halos and populate them with galaxies using a flexible halo occupation framework. We identify halos using the ROCKSTAR phase-space-based halo finder (59), which accurately determines the location of halos and resolves their substructure (60). For our simulations, we identify halos with mass down to $M_{h}≳5\times 10^{10}-2\times 10^{11}M_{⊙}$, depending on the cosmology. We then populate the halos using Halo Occupation Distribution (HOD) models that provide a statistical prescription for populating halos with galaxies based on halo properties such as their mass and concentration. In this work, we use a state-of-the-art HOD model that supplements the standard (61) model with assembly, concentration, and velocity biases. The extra features of our HOD model add additional flexibility that recent works suggest may be necessary to describe galaxy clustering (e.g., refs. 62–64).

Once we have our galaxy distribution in the simulation box, we apply survey realism. We remap the box to a cuboid (65) and then cut out the detailed survey geometry of the BOSS CMASS SGC sample (*Materials and Methods*). This includes masking for bright stars, centerpost, bad field, and collision priority (25, 26, 48). We apply fiber collisions by first identifying all pairs of galaxies within an angular scale of $62^{\prime \prime }$ then, for 60% of the pairs, removing one of the galaxies from the sample. Last, we trim the forward-modeled galaxy catalog to match the 0.45<z<0.6 redshift range and angular range of the observations.

In this work, we analyze a subvolume of BOSS that spans a relatively narrow redshift range, 0.45<z<0.6. Over this range, the number density of the BOSS CMASS galaxy sample does not significantly vary with redshift. We, therefore, use a forward model that is based on N-body simulations at a single z=0.5 snapshot and we do not vary the HOD model with redshift. For a different galaxy sample with a significant redshift dependence, the forward model must be modified to account for the redshift dependence.

**Fig. 1.**
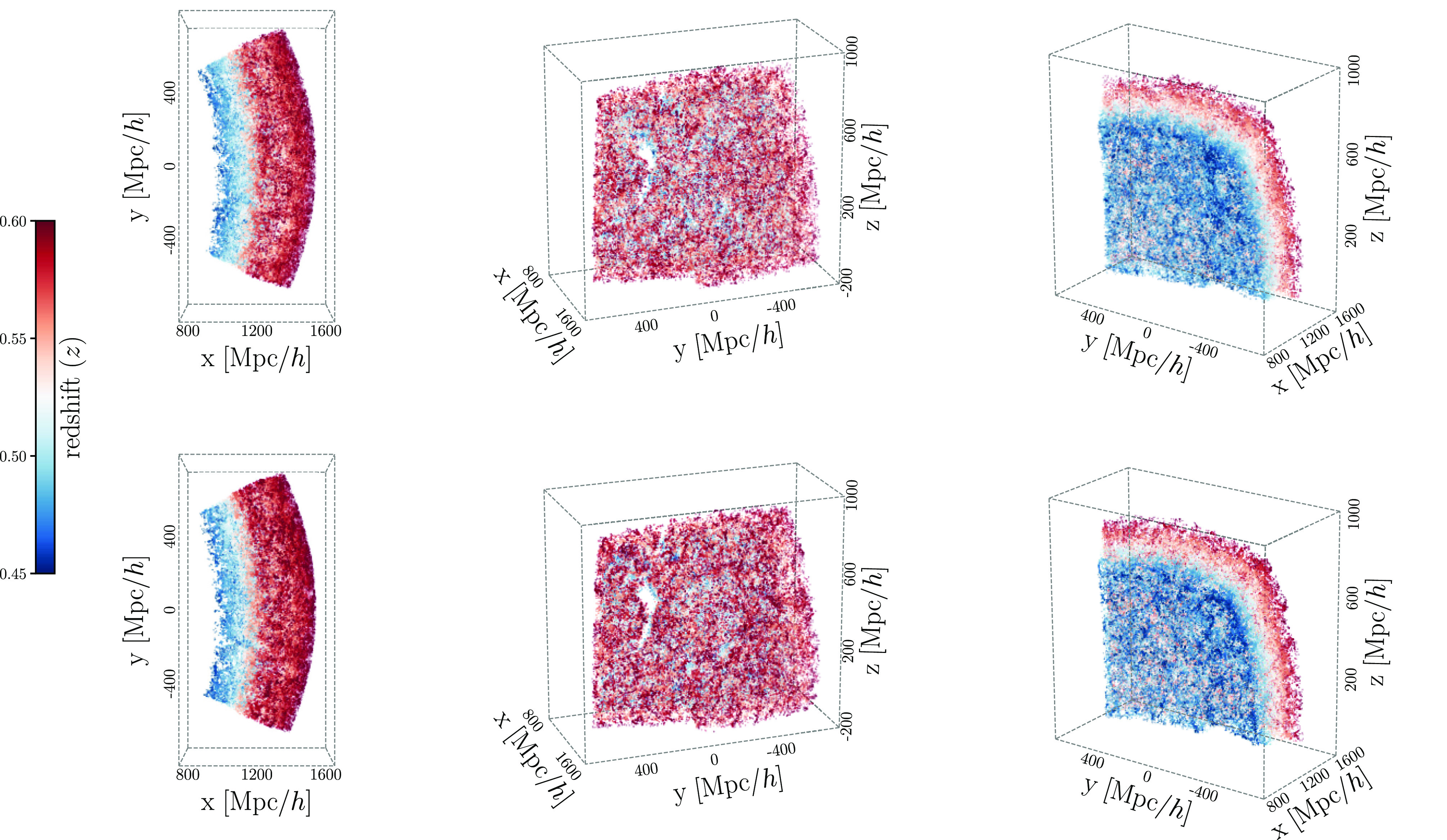
The SimBIG forward model produces simulated galaxy samples with the same survey geometry and observational systematics as the observed BOSS CMASS SGC galaxy sample. We present the three-dimensional (3D) distribution of the galaxies from three different viewing angles. The colormap represents the redshift of the galaxies. In the *Top* set of panels, we present the distribution of galaxies in the CMASS sample. At the *Bottom*, we present the distribution of a simulated galaxy sample, generated from our forward model. The SimBIG galaxy samples are constructed from Quijote N-body dark matter simulations using an HOD model that populates dark matter halos identified using the ROCKSTAR algorithm. The 3D distributions illustrate that our forward model is able to generate galaxy distributions that are difficult to statistically distinguish from observations. For more comparisons of the 3D distributions, we refer readers to 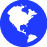
https://youtube.com/playlist?list=PLQk8Faa2x0twK3fgs55ednnHD2vbIzo4z.

**Fig. 2.**
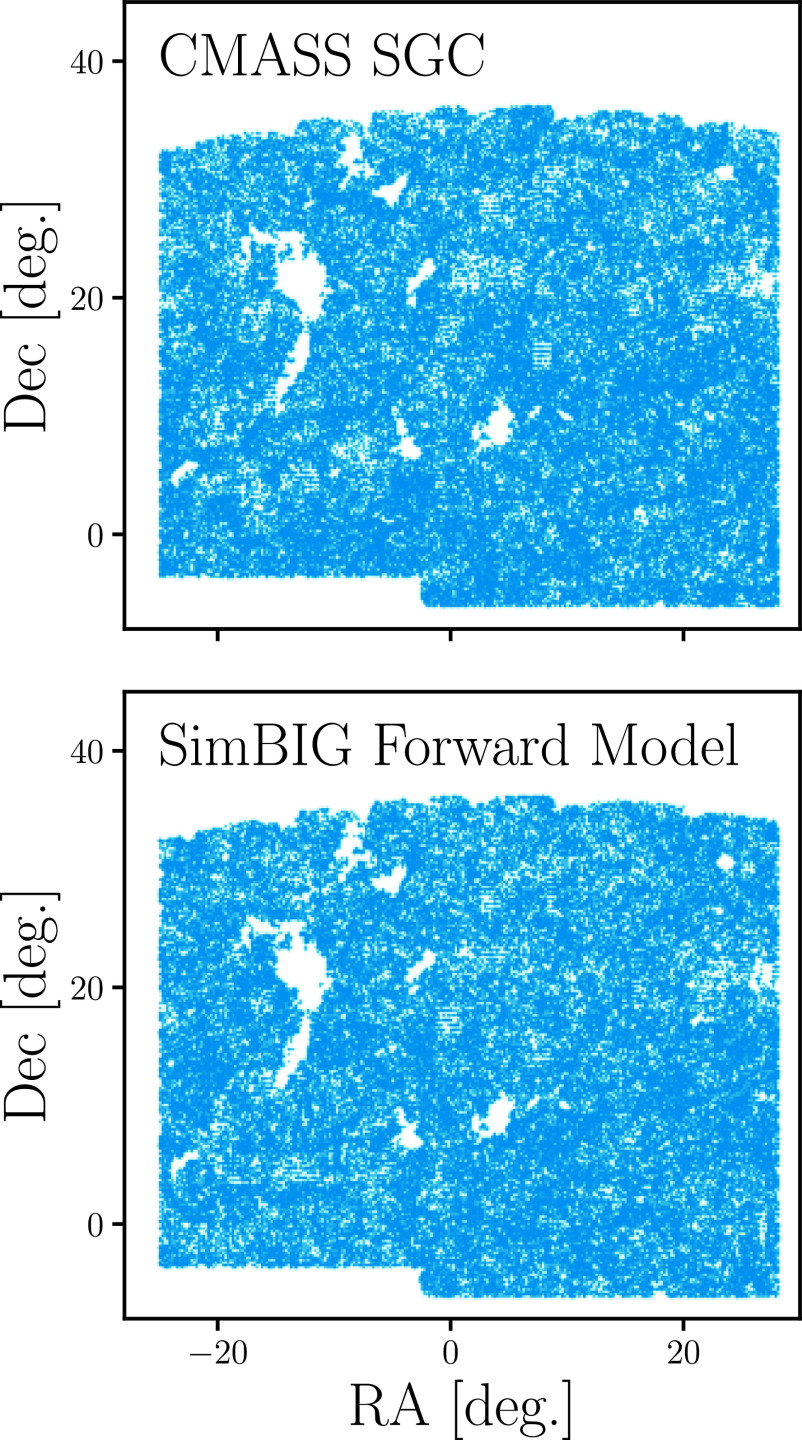
Angular distribution of galaxies from the CMASS sample (*Top*) and a galaxy sample generated using the SimBIG forward model (*Bottom*). Comparison of the angular distributions highlights the detailed CMASS angular selection that we include in our forward model to account for observational systematics.

In total, our forward model has 14 parameters. Five ΛCDM cosmological parameters, $\Omega_{m},\Omega_{b},h,n_{s},\sigma_{8}$, that determine the matter distribution and nine HOD parameters that determine the connection between galaxies and halos. For further details on our forward model, we refer readers to H22b. In the Fig. 1, *Bottom*, we present the 3D spatial distribution of galaxies in our forward model. We present the angular distribution of galaxies in our forward model in Fig. 2. The forward model accurately reproduces the survey geometry and angular footprint of the observed BOSS sample. For additional comparisons of the 3D distributions of galaxies in CMASS and our forward model, we refer readers to 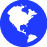
https://youtube.com/playlist?list=PLQk8Faa2x0twK3fgs55ednnHD2vbIzo4z.^*^

### B. Training Dataset for Simulation-Based Inference.

Using our forward model, we construct 20,000 simulated galaxy catalogs. They are constructed from 2,000 QuijoteN-body simulations, each with 10 different sets of HOD parameters, sampled from a broad prior. The N-body simulations are arranged in a Latin Hypercube configuration, which therefore imposes uniform priors on the cosmological parameters that conservatively encompass the Planck cosmological constraints (66).

In principle, SimBIG can be directly applied to the full galaxy catalog if the forward model is capable of accurately modeling observations at all scales. Even with N-body simulations, however, this is not the case due to limitations on mass and time resolution and inadequacies of halo occupation models. Instead, we use summary statistics of the galaxy sample, where we can impose cuts, e.g., based on physical scales, to which our forward model is accurate. Since the primary goal of this work is to present and demonstrate the SimBIG framework, we use the most commonly used summary statistic: the galaxy power spectrum multipole, $P_{\ell }\left(k\right)$. We also include the average galaxy number density of the sample, ${\overset{¯}{n}}_{g}$.

In this work, we use the redshift-space galaxy power spectrum monopole, quadrupole, and hexadecapole (ℓ=0,2, and 4). We measure $P_{0}$, $P_{2}$, and $P_{4}$ for each of the simulated galaxy catalogs using the (67) algorithm. The algorithm accounts for the survey geometry using a random catalog with >4,000,000 randomly positioned objects that have the same radial and angular selection functions as the observed sample. We also include (68) weights with $P_{0}=10^{4}$ to reduce the variance in measured $P_{\ell }$, as is standard practice. We also impose a conservative $k<k_{\max}=0.5\;h/\mathrm{Mpc}$ limit on the $P_{\ell }$, based on the convergence of matter clustering of the Quijote simulations, see H22b or (58) section 5.2 for further details. We also measure $P_{\ell }$ of the BOSS CMASS-SGC galaxy sample with the same algorithm. For the observed ${\hat{P}}_{\ell }\left(k\right)$ we include systematics weights for redshift failures, stellar density, and seeing conditions, which are effects not included in our forward model but shown to be successfully accounted for using the weights (25, 31).

**Fig. 3.**
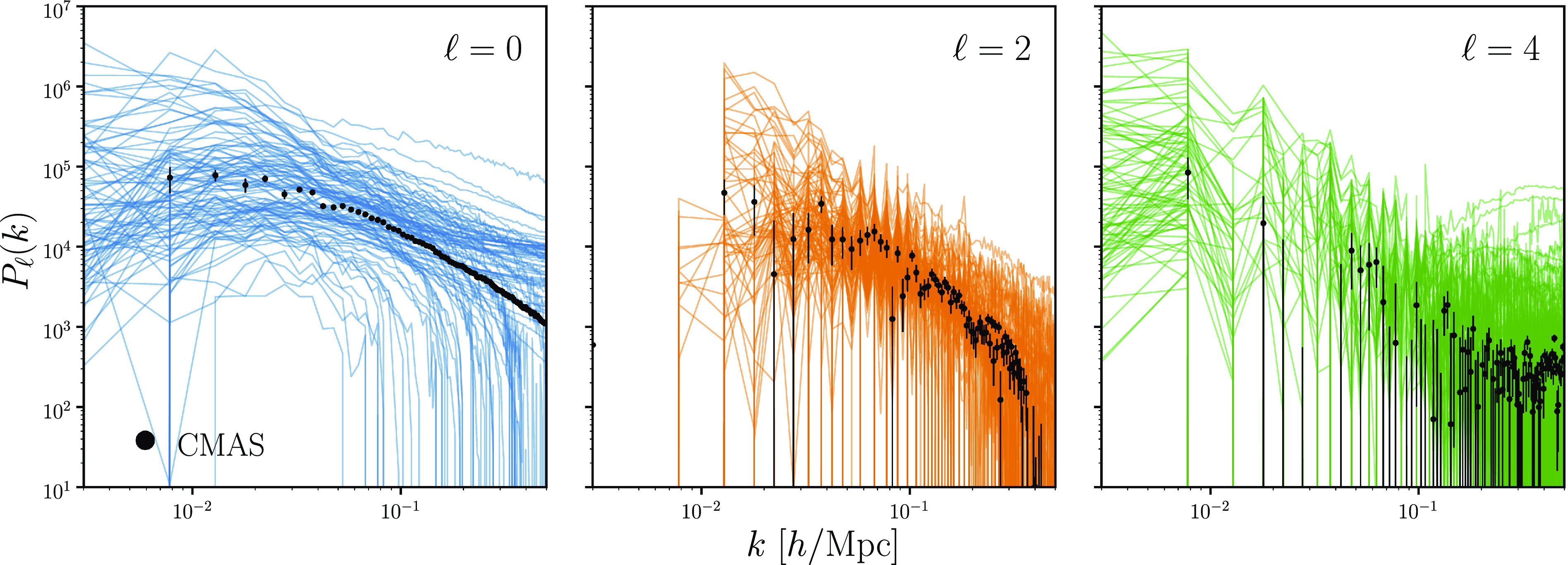
Power spectrum, $P_{\ell }\left(k\right)$, measured from the simulated galaxy catalogs constructed using the SimBIG forward model. We present $P_{\ell }\left(k\right)$ of 100 out of the total 20,000 catalogs for clarity. In each of the panels, we plot the monopole, quadrupole, and hexadecapole of the power spectrum (ℓ=0,2,4). For reference, we include $P_{\ell }\left(k\right)$ measured from the BOSS CMASS SGC galaxy sample (black) with uncertainties estimated from H22b simulations. $P_{\ell }$ is the most commonly used summary statistic of galaxy distribution that measures the two-point clustering. We use $P_{\ell }$ in this work to showcase and validate the SimBIG framework and make detailed comparisons to previous works in the literature. The $P_{\ell }$ of the SimBIG catalogs encompass the BOSS $P_{\ell }$ and, thus, provide a sufficiently broad dataset to conduct SBI.

By using $P_{\ell }$, we can compare the constraints inferred using SimBIG with previous constraints, e.g., refs. 12 and 69 as further validation of SimBIG. To be consistent with previous analyses, we include a nuisance parameter, $A_{\mathrm{shot}}$, that is typically included to account for residual shot noise contribution (e.g., refs. 9, 12, 69). In Fig. 3, we present $P_{\ell }\left(k\right)$ of our forward modeled galaxy catalogs. We randomly select 100 out of the total 20,000 power spectra for clarity. The left, center, and right panels present the monopole, quadrupole, and hexadecapole. Each $P_{\ell }$ and ${\overset{¯}{n}}_{g}$ measurements serve as $x^{\prime }=\left[{\overset{¯}{n}}_{g},P_{\ell }\right]$ in the training dataset $\left\{\left(\theta^{\prime },x^{\prime }\right)\right\}$ for our SBI posterior estimation using normalizing flows.

### C. Simulation-Based Inference with Normalizing Flows.

Normalizing flow models use an invertible bijective transformation, f:z↦θ, to map a complex target distribution to a simple base distribution, π(z), that is fast to evaluate. For SBI, the target distribution is the posterior, p(θ|x), while π(z) is typically a multivariate Gaussian. The transformation f must be invertible and have a tractable Jacobian so that we can evaluate the target distribution from π(z) by change of variables. Since π(z) is easy to sample and evaluate, we can also easily sample and evaluate the target distribution. A neural network with parameters ϕ is used to provide a highly flexible f.

Among the various normalizing flow-based neural density estimators now available in the literature, we use a Masked Autoregressive Flow (MAF; 42). MAF combines normalizing flows with an autoregressive design (70), which is well-suited for estimating conditional probability distributions such as a posterior. A MAF model is built by stacking multiple Masked Autoencoder for Distribution Estimation (MADE; 46) models so that it has the autoregressive structure of MADE models but with additional flexibility to describe complex probability distributions. We use the MAF implementation of the sbi Python package (71, 72).

In training, our goal is to determine the parameters, ϕ, of our normalizing flow, $q_{\phi }\left(\theta \;|\;x\right)$, so that it accurately estimates the posterior, p(θ|x). We can formulate this into an optimization problem of minimizing the Kullback–Leibler (KL) divergence between p(θ,x)=p(θ|x)p(x) and $q_{\phi }\left(\theta \;|\;x\right)p\left(x\right)$, which measures the difference between the two distributions.

[1] $$\begin{array}{cc} \min_{\phi }D_{\mathrm{KL}} & (p\left(\theta ,x\right)\|q_{\phi }\left(\theta \;|\;x\right)p\left(x\right))\\ & =\min_{\phi }\int p\left(\theta ,x\right)\log\frac{p(\theta \;|\;x)}{q_{\phi }\left(\theta \;|\;x\right)}\;d\theta dx, \end{array}$$

[2] $$\begin{array}{cc} & \approx \min_{\phi }\sum_{i}\log p\left(\theta_{i}\;|\;x_{i}\right)-\log q_{\phi }\left(\theta_{i}\;|\;x_{i}\right), \end{array}$$

[3] $$\begin{array}{cc} & \approx \min_{\phi }\sum_{i}-\log q_{\phi }\left(\theta_{i}\;|\;x_{i}\right), \end{array}$$

[4] $$\begin{array}{cc} & \approx \max_{\phi }\sum_{i}\log q_{\phi }\left(\theta_{i}\;|\;x_{i}\right). \end{array}$$

Eq. **2** follows from the fact that the training dataset $\left\{\left(\theta^{\prime },x^{\prime }\right)\right\}$ is constructed by sampling from p(θ,x) with our forward model.

We split the training data into a training and validation set with a 90/10 split, then maximize Eq. **4** over the training set. We use the ADAM optimizer (73) with a learning rate of $5\times 10^{-4}$. We prevent overfitting by stopping the training when the log-likelihood Eq. **4** evaluated on the validation set fails to increase after 20 epochs. We determine the architecture of our normalizing flow through experimentation. Our final trained model has 6 MADE blocks, each with 9 hidden layers and 186 hidden units. For further details on the training procedure, we refer readers to H22b. Once trained, we estimate the posterior of our 5 cosmological, 9 HOD parameters, and 1 nuisance parameter for the BOSS CMASS SGC by sampling our normalizing flow: $q_{\phi }\left(\theta \;|\;\hat{x}=\left[{\hat{\overset{¯}{n}}}_{g},\hat{P_{\ell }}\right]\right)$. $\hat{P_{\ell }}$ and ${\hat{\overset{¯}{n}}}_{g}$ represent the $P_{\ell }$ and ${\overset{¯}{n}}_{g}$ measurements for the observed BOSS CMASS SGC sample.

## 1. Results

**Fig. 4.**
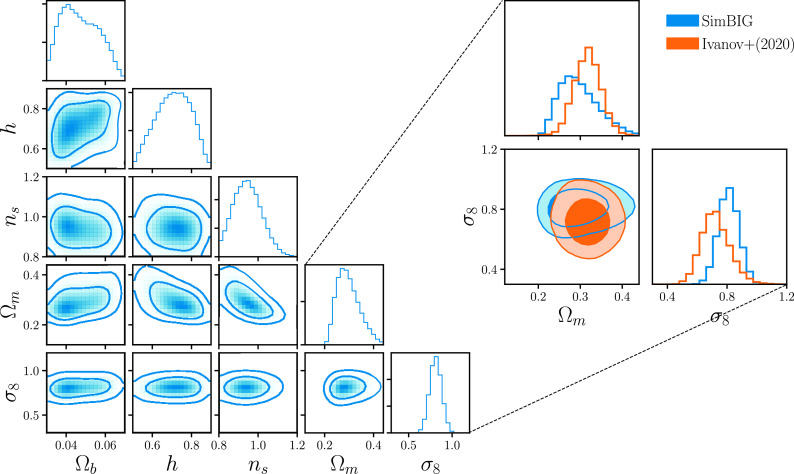
(*Left*) Posterior of cosmological parameters inferred from $P_{\ell }$ using SimBIG. In the diagonal panels, we present the marginalized one-dimensional (1D) posterior of each parameter. The other panels present the 2D posteriors that illustrate the degeneracies between two parameters. The contours mark the 68 and 95 percentiles. We accurately analyze $P_{\ell }$ down to nonlinear regimes, $k_{\max}=0.5\;h/\mathrm{Mpc}$, by using a simulation-based forward model that includes observational systematics. (*Right*) We focus on the posteriors of ($\Omega_{m}$, $\sigma_{8}$), the parameters that can be most significantly constrained by galaxy clustering alone. We derive $\Omega_{m}=0.292_{-0.040}^{+0.055}$ and $\sigma_{8}=0.812_{-0.068}^{+0.067}$. Our $\sigma_{8}$ constraints are 27% tighter than the (12) $k_{\max}=0.25\;h/\mathrm{Mpc}$ PT constraint (orange).

We present the posterior distribution of the ΛCDM cosmological parameters, $\Omega_{m},\Omega_{b},h,n_{s},\sigma_{8}$, inferred from $P_{\ell }\left(k\right)$ using SimBIG in Fig. 4. The posterior is inferred from the BOSS $P_{\ell }\left(k\right)$ down to $k_{\max}=0.5\;h/\mathrm{Mpc}$. The diagonal panels present the marginalized one-dimensional posteriors for each parameter. The other panels present marginalized two-dimensional posteriors of different parameter pairs that highlight parameter degeneracies. We mark the 68 and 95 percentiles of the posteriors with the contours. We infer the posterior of HOD and nuisance parameters; however, we do not include them in the figure for clarity. Among the cosmological parameters, the SimBIG posterior significantly constrains $\Omega_{m}$ and $\sigma_{8}$. This is consistent with previous works that relied on priors from Big Bang nucleosynthesis or cosmic microwave background (CMB) experiments for the other parameters, e.g., refs. 12 and 15. We infer $\Omega_{m}=0.292_{-0.040}^{+0.055}$ and $\sigma_{8}=0.812_{-0.068}^{+0.067}$.

In the accompanying paper H22b, we present the detailed validation of the SimBIG posterior using a suite of 1,500 test simulations. We construct the test suite using different forward models than the one used for our training data. They are constructed using different N-body simulations, halo finders, and HOD models. This is to ensure that the cosmological constraints we derive are independent of the choices and assumptions made in our forward model. Then, we conduct a “mock challenge” where we infer posteriors of the cosmological parameters for each of the test simulations. Since we know the true cosmological parameter values of the test simulations, we can assess both the accuracy and precision of the inferred posteriors.

H22b reveals that SimBIG produces unbiased posteriors. On the other hand, the posteriors are conservative, i.e., they are broader than the true posterior. This is due to the limited number of N-body simulations used to construct our training dataset. Although we use 20,000 forward-modeled simulations, they are constructed from 2000 N-body simulations with different values of cosmological parameters. This makes our estimate of the KL divergence, Eq. **4** noisy and makes training the SimBIG normalizing flow more challenging. Our constraint on $\Omega_{m}$ is particularly conservative. Additional N-body simulations or improvements to the training would significantly improve the precision of our posteriors.

Despite the fact that they are conservative, the $\sigma_{8}$ posterior from SimBIG is significantly more precise than constraints from previous works. Mikhail *et al.* (12) analyzed the $P_{\ell }$ of the BOSS CMASS galaxy sample using the PT approach with an analytic model based on effective field theory. For the CMASS SGC sample, with uniform priors on the cosmological parameters, and with $k_{\max}=0.25\;h/\mathrm{Mpc}$, Mikhail *et al.* (12) inferred $\sigma_{8}=0.719_{-0.085}^{+0.100}$. With SimBIG, we improve $\sigma_{8}$ constraints by 27% over the standard galaxy clustering analysis. We emphasize that this improvement is roughly equivalent to analyzing a galaxy survey ∼60% larger than the original survey using PT.

Recently, Kobayashi *et al.* (15) also analyzed the $P_{\ell }$ of BOSS CMASS sample but using a theoretical model based on a halo power spectrum emulator. Instead of using a galaxy bias scheme used by PT to connect the galaxy and matter distributions, Kobayashi *et al.* (15) used halo power spectra predicted by an emulator and a halo occupation framework, similar to the HOD model in our forward model. We note that while the halo power spectrum emulator is trained using simulations, the approach in ref. 15 does not forward model observational systematics. They also make the same assumptions on the form of the likelihood as PT analyses for their inference. For the CMASS SGC sample, with uniform priors on all cosmological parameters, and with $k_{\max}=0.25\;h/\mathrm{Mpc}$, Kobayashi *et al.*(15) inferred $\sigma_{8}=0.790_{-0.072}^{+0.083}$. Kobayashi *et al.* (15) constraints are tighter than Mikhail *et al.* (12) PT constraints because the halo occupation model provides a more compact framework for modeling galaxies. Nevertheless, with SimBIG, we improve on their $\sigma_{8}$ constraints by 13%.

SimBIG produces significantly tighter constraints on $\sigma_{8}$ because we are able to accurately extract cosmological information available on small, nonlinear, scales. With our forward modeling approach, we can accurately model nonlinear clustering and robustly account for observational systematics down to $k_{\max}=0.5\;h/\mathrm{Mpc}$. In both refs. 12 and 15, they restrict their analysis to $k_{\max}<0.25\;h/\mathrm{Mpc}$ due to the limitations of their analyses on smaller scales.

**Fig. 5.**
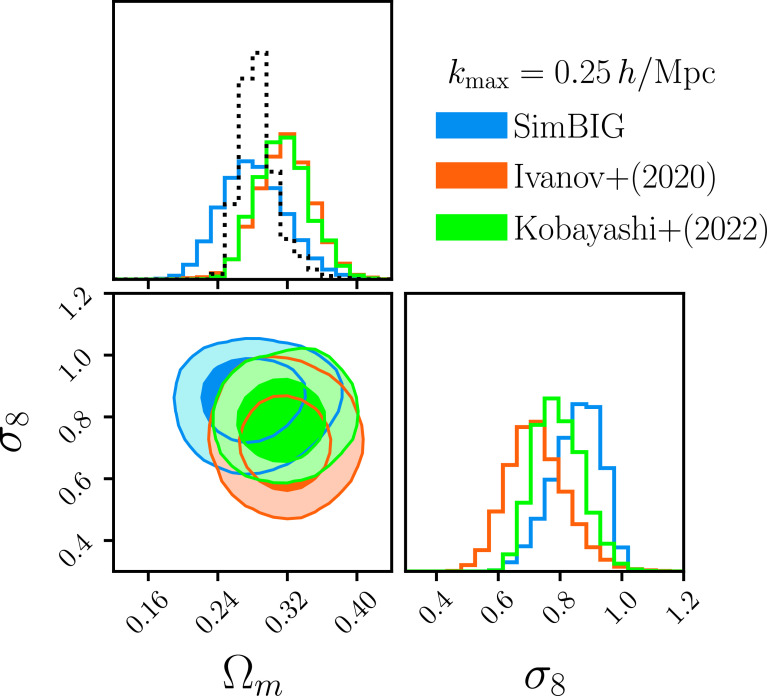
Comparison of the ($\Omega_{m}$, $\sigma_{8}$) posteriors inferred from the $P_{\ell }$ CMASS-SGC analysis for $k_{\max}=0.25\;h/\mathrm{Mpc}$ from SimBIG (blue), the ref. 12 PT approach (orange), and the ref. 15 emulator approach (green). The contours represent the 68 and 95 percentiles. We find overall good agreement among the posteriors. For $\Omega_{m}$, SimBIG infers consistent but broader posterior due to the fact that we use a limited number of simulations. We estimate the expected $\Omega_{m}$ constraints with more simulations using posterior “recalibration” (black dotted). For $\sigma_{8}$, both SimBIG and ref. 15 derive tighter constraints than ref. 12 due to the fact that we use halo occupation instead of a galaxy bias prescription. Meanwhile, the precision of our $\sigma_{8}$ constraint is in excellent agreement with ref. 15, which uses a similar halo occupation model. The consistency of the $k_{\max}=0.25\;h/\mathrm{Mpc}$ posteriors demonstrate that the improvements in the $k_{\max}=0.5\;h/\mathrm{Mpc}$ constraints come from additional cosmological information in the nonlinear regime that SimBIG can robustly extract.

To further verify that our improvement comes from constraining power on small scales, we analyze $P_{\ell }$ to $k_{\max}=0.25\;h/\mathrm{Mpc}$ using SimBIG. In Fig. 5, we present the SimBIG$k_{\max}=0.25\;h/\mathrm{Mpc}$ posterior (blue) along with the posteriors from (12, orange) and (15, green). We focus our comparison on $\Omega_{m}$ and $\sigma_{8}$, the cosmological parameters that can be most competitively constrained with galaxy clustering alone. The contours again represent the 68 and 95 percentiles. We find overall good statistical consistency among the posteriors. For $\sigma_{8}$, our $k_{\max}=0.25\;h/\mathrm{Mpc}$ places a $\sigma_{8}=0.861_{-0.091}^{+0.070}$ constraint. This is significantly broader than our $k_{\max}=0.5\;h/\mathrm{Mpc}$ constraint and, thus, demonstrates that the constraining power is in fact from nonlinear scales. Furthermore, the precision of the $k_{\max}=0.25\;h/\mathrm{Mpc}$ SimBIG constraint is in excellent agreement with the ref. 15 constraint. This serves as further validation of SimBIG, since ref. 15 uses a similar halo occupation framework to model the power spectrum.

For $\Omega_{m}$, we infer broader posteriors than refs. 12 and 15. As we discuss above and in H22b, this is due to the fact that the SimBIG normalizing flow is trained using a limited number of simulations. To estimate the expected improvement in the $\Omega_{m}$ constraints without this limitation, we use the posterior “re-calibration” procedure from ref. 74. The recalibration uses the posteriors inferred for the test simulations and their true parameter values. We calculate the local probability integral transform (75), a diagnostic of the inferred posteriors, and use this quantity to derive a weighting scheme that corrects the posteriors so that it matches the true posterior of the test simulations.

The recalibration uses test simulations, so we do not use it for inference. However, it provides a bound for the SimBIG constraints if we were to have sufficient training simulations. The recalibrated posterior constrains $\Omega_{m}=0.284_{-0.017}^{+0.021}$. For reference, we mark the recalibrated $\Omega_{m}$ constraint (black dotted) in Fig. 5. The recalibrated $\Omega_{m}$ is in good agreement with both the refs. 12 and 15 constraints. It is significantly tighter than the original SimBIG constraint and illustrates that additional training simulations would significantly improve the precision of the SimBIG$\Omega_{m}$ constraints.

Based on our $k_{\max}=0.5\;h/\mathrm{Mpc}$ posterior, we infer $S_{8}=\sigma_{8}\sqrt{\Omega_{m}/0.3}=0.802_{-0.092}^{+0.102}$ (and $0.797_{-0.076}^{+0.078}$ for our recalibrated posterior). Multiple recent large-scale structure studies have reported a “$S_{8}$ tension” with constraints from ref. 66 CMB analysis. They find significantly lower values of $S_{8}$ than Planck (23, 76–82). PT analyses of BOSS also infer relatively low values of $S_{8}$. Ref. 12, for instance, infers $S_{8}=0.737_{-0.092}^{+0.110}$. This $S_{8}$ tension between constraints from large-scale structure and CMB analyses has motivated a number of works to explore modifications of the standard ΛCDM cosmological model, e.g., refs. 83–86. We do not find a significant $S_{8}$ tension with the Planck constraints (66). However, given the statistical precision of our $S_{8}$ constraint, we refrain from more detailed comparison and discussion.

## 2. Conclusions

We present SimBIG, a forward modeling framework for analyzing galaxy clustering using SBI. As a demonstration of the framework, we apply it to the BOSS CMASS SGC, a galaxy sample at z∼0.5. We analyze the galaxy power spectrum multipoles ($P_{\ell }$), the most commonly used summary statistic of the galaxy spatial distribution, to showcase and validate the SimBIG framework.

SimBIG utilizes a full forward model of the CMASS sample, unlike standard approaches that use analytic models of the summary statistic. The forward model is based on high-resolution QuijoteN-body simulations that can accurately model the matter distribution on small scales. It uses halo modeling and a state-of-the-art HOD model with assembly, concentration, and velocity biases that provide a flexible mapping between the matter and galaxy distributions. The forward model also includes realistic observational systematics such as survey geometry and fiber collisions. With this forward modeling approach, we can leverage the predictive power of simulations to analyze small, nonlinear, scales as well as higher-order clustering. It also provides a framework to account for systematics for any summary statistic.

Using the forward model, we construct 20,000 simulated CMASS-like samples that span a wide range of cosmological and HOD parameters. We measure $P_{\ell }$ and ${\overset{¯}{n}}_{g}$ for each of these samples and use the measurements as the training dataset for SBI. To estimate the posterior, we use neural density estimation based on normalizing flows. Using the training dataset, we train our normalizing flows by minimizing the KL divergence between its posterior estimate and the true posterior. Once trained, we apply our normalizing flow to the observed summary statistics to infer the posterior of 5 cosmological, 9 HOD, and 1 nuisance parameter.

Focusing on the cosmological parameters, we derive significant constraints on: $\Omega_{m}=0.292_{-0.040}^{+0.055}$ and $\sigma_{8}=0.812_{-0.068}^{+0.067}$. Our $\sigma_{8}$ constraints are 27% tighter than the ref. 12 constraints using a standard PT approach on the same galaxy sample. This improvement is roughly equivalent to increasing the volume of the galaxy survey by ∼60% for a standard PT $P_{\ell }$ analysis. The SimBIG constraints are inferred from $P_{\ell }$ out to $k_{\max}=0.5\;h/\mathrm{Mpc}$ while the PT constraints are limited to $k_{\max}=0.25\;h/\mathrm{Mpc}$. The improvement is driven by the additional cosmological information on nonlinear scales that SimBIG can robustly extract.

We also infer the posterior using SimBIG from $P_{\ell }$ with $k_{\max}=0.25\;h/\mathrm{Mpc}$ and compare it to posteriors in the literature. In particular, the SimBIG$\sigma_{8}$ constraint for $k_{\max}=0.25\;h/\mathrm{Mpc}$ are in excellent agreement with the constraint from the recent halo model–based emulator analysis of ref. 15. Since they use a similar halo occupation framework as SimBIG, this comparison firmly verifies the robustness of our constraints. The comparison also confirms that the improvement in SimBIG$k_{\max}=0.5\;h/\mathrm{Mpc}$ constraints come from the nonlinear regime. In the accompanying H22b, we present additional tests of SimBIG through a mock challenge. The tests use a suite of 1,500 test simulations constructed with different forward models to demonstrate that SimBIG produces unbiased cosmological constraints. H22b also presents further details on our forward model and discusses posterior constraints on HOD parameters.

SimBIG can also extract higher-order cosmological information. Standard galaxy clustering analyses primarily focus on two-point clustering statistics. Analyses of higher-order statistics have been limited to, e.g., the bispectrum (18, 19, 87), and even these analyses extract only limited cosmological information beyond linear scales. In subsequent work, we will use SimBIG to analyze the BOSS CMASS galaxies using higher-order statistics (the bispectrum) and nonstandard observables that contain additional cosmological information: e.g., marked power spectrum, skew spectra, void probability functions, and wavelet- scattering-like statistics. We will also apply SimBIG to analyze field-level summary statistics that capture all the information in the galaxy field using convolutional and graph neural networks.

SimBIG can also be extended to upcoming spectroscopic galaxy surveys observed using DESI, PFS, Euclid, and Roman, which will probe huge cosmic volumes over the next decade. They will produce the largest and most detailed three-dimensional maps of galaxies in the Universe. These surveys are already expected to provide the most precise constraints on cosmological parameters using standard analyses. SimBIG can further exploit the statistical power of these surveys to place even tighter constraints on cosmological parameters and produce the most stringent tests of the standard ΛCDM cosmological model and beyond.

## 3. Materials and Methods

### Observations: SDSS-III BOSS.

In this work, we analyze observations from the Sloan Digital Sky Survey SDSS-III (47, 48) Baryon Oscillation Spectroscopic Survey (BOSS) Data Release 12. In particular, we use the CMASS galaxy sample, which selects high stellar mass Luminous Red Galaxies (LRGs) over the redshift 0.43<z<0.7 (88). We restrict our analysis to CMASS galaxies in the Southern Galactic Cap (SGC) and impose a redshift cut of 0.45<z<0.6 and the following angular cuts: Dec>−6 and 28>RA>−25. In upper panels in Fig. 1, we present the three-dimensional distributions of our CMASS SGC galaxy sample at three different viewing angles. We also present the angular distribution of the sample in Fig. 2. In total, our CMASS SGC galaxy sample contains 109,636 galaxies.

## Supplementary Material

Appendix 01 (PDF)

Movie S1. In **anim_slices_4.mov**, we present the projected slices of the galaxy distribution along the radial direction from the point of the view of an observer. The top and bottom panels present the BOSS CMASS and simulated galaxy samples respectively. The right panels highlight the progression of the radial slice for both samples.

Movie S2. In **anim_slices_1.mov**, we present projected slice of the galaxy distribution along the *Z* axis for the BOSS CMASS (top) and simulated (bottom) galaxy samples. The panels on the right highlight the progression of the *Z* slice for both samples.

Movie S3. In **anim_3d_rot.mov**, we present a rotating view with of the galaxy distribution with the BOSS CMASS and simulated galaxy samples on the left and right panels, respectively. The color indicates galaxy redshift.

## Data Availability

Dataset data have been deposited in Zenodo (https://doi.org/10.5281/zenodo.8221749) (89).

## Footnotes

^*^https://youtube.com/playlist?list=PLQk8Faa2x0twK3fgs55ednnHD2vbIzo4z.
