# Supplementary material for: A forward modeling approach to analyzing galaxy clustering with SimBIG
Source: Proc Natl Acad Sci U S A. 2023 Oct 11;120(42):e2218810120. doi: 10.1073/pnas.2218810120 (PMC10589614; doi:10.1073/pnas.2218810120)
Supplement: Supplementary file 1 — Appendix 01 (PDF) [file pnas.2218810120.sapp.pdf]

Legend for supporting videos

The three-dimensional distribution of galaxies encodes cosmological information that can be used to constrain the nature of dark matter and dark energy and measure the contents of the Universe.
With the `SimBIG` forward model and simulation-based inference we can directly extract this information from galaxy surveys, such as BOSS, down to small non-linear scales.
In the supporting videos, we compare a simulated galaxy sample constructed from our `SimBIG` forward model to the observed BOSS CMASS SGC galaxy sample.

In **anim_3d_rot.mov**, we present a rotating view with of the galaxy distribution with the BOSS CMASS and simulated galaxy samples on the left and right panels, respectively.
The color indicates galaxy redshift.

In **anim_slices_1.mov**, we present projected slice of the galaxy distribution along the Z axis for the BOSS CMASS (top) and simulated (bottom) galaxy samples.
The panels on the right highlight the progression of the Z slice for both samples.

In **anim_slices_4.mov**, we present the projected slices of the galaxy distribution along the radial direction from the point of the view of an observer.
The top and bottom panels present the BOSS CMASS and simulated galaxy samples respectively.
The right panels highlight the progression of the radial slice for both samples.
